# Gastrointestinal Manifestations of COVID-19: A Review of What We Know

**DOI:** 10.31486/toj.20.0086

**Published:** 2021

**Authors:** Andrew Groff, Madison Kavanaugh, Devyani Ramgobin, Brendan McClafferty, Chander Shekher Aggarwal, Reshma Golamari, Rohit Jain

**Affiliations:** ^1^ Penn State College of Medicine, Hershey, PA; ^2^ Boonshoft School of Medicine, Wright State University, Dayton, OH; ^3^ Touro College of Osteopathic Medicine, Middletown, NY; ^4^ Lake Erie College of Osteopathic Medicine, Erie, PA; ^5^ University of Medical Sciences, Delhi, India; ^6^ Department of Internal Medicine, Penn State Health Milton S. Hershey Medical Center, Hershey, PA

**Keywords:** *Coronavirus*, *COVID-19*, *disease transmission–infectious*, *gastrointestinal tract*, *liver*, *SARS-CoV-2*

## Abstract

**Background:** The coronavirus disease 2019 (COVID-19) is not just a disease of the respiratory system. The virus can affect the gastrointestinal (GI) tract as well. Recognizing the various manifestations in every organ system is important because these manifestations can contribute to community-based transmission.

**Methods:** We outline the evidence of the pathophysiology of COVID-19 in the GI tract, the effects of the virus on the gut and liver, the presence of the virus in stool samples, and the potential for fecal-oral transmission of COVID-19. Most of the literature sources used in this paper are case studies from China following the surge of COVID-19 infection.

**Results:** In patients with COVID-19, GI symptoms such as anorexia, nausea, vomiting, diarrhea, and abdominal pain have presented in conjunction with respiratory symptoms such as fever, shortness of breath, and cough. Evidence also shows acute hepatocellular injury, indicated by elevated liver enzymes such as alanine aminotransferase, aspartate aminotransferase, and gamma-glutamyl transferase. Fecal-oral transmission of COVID-19 is suspected because of the presence of COVID-19 RNA in stool samples of COVID-19–positive patients.

**Conclusion:** Even without the presence of respiratory symptoms, several GI symptoms are associated with COVID-19 infection, as well as possible fecal-oral transmission. Therefore, COVID-19 infection should be considered for patients presenting with primarily GI symptoms.

## INTRODUCTION

The virus responsible for the coronavirus disease pandemic and global health emergency that began in 2019 is the severe acute respiratory syndrome coronavirus 2 (SARS-CoV-2), a positive-sense, single-stranded RNA virus and member of the *Betacoronavirus* genus, specifically in lineage B. The rapid transmission of this virus across the globe has resulted in widespread infection, morbidity, and mortality, with a reported case fatality rate as of November 2020, ranging from 2.3% in the United States to 4.9% in Italy.^1-6^

The respiratory illness caused by SARS-CoV-2 is commonly referred to as COVID-19. Typically reported symptoms of COVID-19 include fever, nonproductive cough, myalgia, malaise, and dyspnea.^7-9^ Less frequent symptoms include headache, productive cough, rhinorrhea, and hemoptysis. In addition, abdominal pain, diarrhea, nausea, vomiting, and other enteric symptoms are prevalent in some patients. In one study of 204 COVID-19–positive patients in China, 50.5% reported gastrointestinal (GI) symptoms, including lack of appetite, diarrhea, vomiting, nausea, and abdominal pain.^10^ Case reports and case series have also documented patients with COVID-19 who presented with GI symptoms early in the disease course.^11-14^ Further investigations demonstrated that the liver may be affected by COVID-19.^15,16^

## PATHOPHYSIOLOGY OF COVID-19 AND THE GASTROINTESTINAL TRACT

The leading hypothesis for the mechanism of transmission of SARS-CoV-2 is through aerosolized respiratory droplets.^3^ When an individual comes into contact with the pathogen, the virus binds to the angiotensin-converting enzyme 2 (ACE2) receptors in the lungs.^17^ The spike glycoprotein of SARS-CoV-2 attaches to the ACE2 receptor and enables efficient viral entry into the cells, leading to viral replication and spread throughout the body.^18^ The intestinal (principally absorptive enterocytes of the ileum and colon) and esophageal epithelium also highly express ACE2 receptors. Further, the glandular cells of both the stomach and duodenum are reported to express ACE2,^19^ so SARS-CoV-2 may be able to infect intestinal epithelial cells via ACE2 receptors. The ACE2 receptors in the GI tract maintain a regulatory role in amino acid homeostasis, gut microbiome, and innate immunity.^20^ Consequently, the binding of SARS-CoV-2 to ACE2 receptors in the GI tract may result in GI symptoms such as abdominal pain and diarrhea. Of note, ACE2 appears to be more highly expressed in patients with preexisting colorectal cancer or adenomas compared to healthy controls.^21^ However, whether patients with GI cancers such as colorectal carcinoma are at higher risk of being infected is still unknown, so further study of GI cancers and COVID-19 infection rates and outcomes is warranted.

Despite clinical studies demonstrating liver injury concurrent with COVID-19 infection in patients in intensive care units (ICUs) in Wuhan, China, primarily via elevated alanine aminotransferase (ALT) and aspartate aminotransferase (AST) levels, a clear mechanism of liver injury has not been elucidated.^7^ However, proposed mechanisms for liver injury include hepatic tropism for SARS-CoV-2 as well as direct cytopathic effects based on studies demonstrating in situ hybridization analysis demonstrating SARS-CoV-2 virions in vessel lumens and endothelial cells of portal veins in liver specimens of infected persons. In addition, a study involving liver samples from 2 deceased infected persons with elevated liver enzymes via electron microscopic analysis showed the presence of intact viral particles in the cytoplasm of hepatocytes.^22^ A study published as a preprint in February 2020 reported that ACE2 receptors are highly expressed in cholangiocytes (59.7% of cells) but less expressed in hepatocytes (2.6% of cells).^23^ Although cholangiocytes are the predominant cells to express ACE2 receptors, a hepatocellular pattern of injury with mild to moderate ALT and AST elevations is more common than a cholestatic pattern of injury in reported COVID-19 infections.^23^ Acute acalculous cholecystitis has been associated with viral infections in pediatric patients, and while the exact pathogenesis is unknown, evidence shows direct invasion of the gallbladder by the hepatitis A virus.^24^ Similarly, Musa hypothesized that COVID-19 causes liver dysfunction via direct viral cytotoxicity from binding of similar ACE2 receptors found in the respiratory and GI tracts.^25^ An estimated 14.8% to 78% of infected individuals develop some form of liver injury, primarily mild to moderate elevation in ALT and AST levels.^1,25-27^ Elevated levels of total bilirubin are also found in 6.1% to 18% of COVID-19–positive patients.^16,26-27^ In these studies, patients with severe forms of COVID-19 requiring ICU care exhibited a higher prevalence of ALT, AST, and total bilirubin elevations compared to patients with nonsevere COVID-19 cases. Furthermore, a 2020 study of liver injury in COVID-19 showed that gamma-glutamyl transferase levels were elevated in 54% of patients.^15^ In most cases of COVID-19, transient elevation of liver enzymes occurs and improves as the patient recovers.^28^

## FECAL-ORAL TRANSMISSION: COVID-19 DETECTION IN STOOL SAMPLES

Little definitive evidence supports fecal-oral transmission of COVID-19. Still, the possibility exists, as 29% to 55% of infected patients have detectable COVID-19 RNA in their stool samples.^10,29,30^ However, the presence of COVID-19 RNA or live virus in stool samples does not correlate with the presence or absence of GI symptoms.^10^ Overall, the diagnostic accuracy of stool or anal swabs is almost as accurate as nasopharyngeal swabs in the detection of COVID-19.^31,32^ Ling et al reviewed the reverse transcription polymerase chain reaction results for oropharyngeal swabs, stool, urine, and serum samples for 292 confirmed COVID-19 cases.^33^ The analysis of these laboratory results demonstrated that viral RNA can be detected in the stool of 82% of patients even after testing negative via respiratory sampling. If viral RNA is detectable after respiratory sampling is negative, patients could possibly transmit the virus to others via fecal shedding for up to 5 weeks after negative respiratory samples.^34^ Prolonged fecal shedding of viral RNA and live virus may have clinical implications. If definite evidence shows fecal-oral transmission of COVID-19, health care workers must practice extreme caution when handling stool samples and caring for infected individuals, even if COVID-19 nasopharyngeal swab testing is negative.

## GASTROINTESTINAL MANIFESTATIONS OF COVID-19

Many patients with COVID-19 present with GI symptoms and with pneumonia-like illness with symptoms such as fever, cough, and dyspnea.^10-14^ GI symptoms are wide ranging and include nausea, vomiting, abdominal pain, diarrhea, and anorexia. The prevalence of these general GI symptoms has been reported to vary between 3% and 79% in patients with confirmed COVID-19.^35-38^ According to a review published in March 2020 by Tian et al, anorexia was the most commonly reported GI symptom in adults, occurring in 39.9% to 50% of confirmed cases.^35^ The next most common symptom was diarrhea, reported for 2% to 49.5% of patients.^35^ The prevalence of nausea and vomiting ranged between 1% and 29.4% in COVID-19–positive adults.^35^ Abdominal pain has been less reported in the literature, with prevalence ranging between 2.2% and 6% of patients with confirmed COVID-19.^10,35,37^

Among children, Bolia et al noted that 12% of pediatric patients (n=9) with COVID-19 infection had GI manifestations, including clinical signs of pseudoappendicitis.^39^ Bolia et al also reported that children infected with COVID-19 had GI symptoms more often than adults with COVID-19, particularly in cases of multisystem inflammatory syndrome, in which 84% of patients had GI symptoms (n=44).^39^ However, Mao et al reported that pediatric patients have similar prevalence of GI symptoms compared to adults, with children having similar risk of liver injury compared to adults but less likelihood of having elevated liver enzymes.^40^

Diarrhea has emerged as an early symptom of COVID-19 because of its prevalence in otherwise asymptomatic COVID-19 patients.^37^ Reports suggest that diarrhea presents between 1 and 8 days after infection onset, with a mean onset of 3.3 days.^35^ In a cohort of 308 patients from Wuhan, China, diarrhea was present in 44.7% of confirmed COVID-19 cases, while in another cohort of 138 patients from the same area, diarrhea was reported in 10.8% of confirmed cases.^10,37^ A study of 1,099 patients from 552 hospitals in China reported that 3.8% of patients with confirmed COVID-19 had diarrhea.^1^ Liang et al showed that the high expression of the ACE2 receptor in the small intestine has a direct impact on inflammation and the development of diarrhea and also showed a statistically significant difference in the prevalence of diarrhea (between 2% and 33%) in patients with confirmed COVID-19 in 3 hospitals in Wuhan, China (*P*=0.016).^14^ These data suggest that the lack of precise criteria to define diarrhea leads to the disparity in diarrhea reporting for patients with COVID-19 and that the variability in prevalence figures warrants further investigation. In an analysis of 116 patients with COVID-19 infection in the United States, 31.9% reported GI manifestations such as loss of appetite, nausea, vomiting, and diarrhea.^41^ These symptoms were not the initial presenting symptoms but appeared throughout the course of infection.

Awareness of the time course of GI symptoms is crucial because these symptoms may be one of the first signs of COVID-19 infection. A small case series from China reported 9 patients who initially presented with GI symptoms only, and 4 of the 9 patients did not develop fever or respiratory symptoms.^42^ The first confirmed COVID-19 patient in the United States initially presented to the hospital because of nausea and vomiting and not because of fever or dyspnea.^43^ Other case reports suggest that nausea, diarrhea, and abdominal pain present at the onset of COVID-19 infection, followed by fever and dyspnea a few days later.^10,12^ The presence of early GI symptoms may make the diagnosis of COVID-19 difficult because clinicians may be misled. However, these symptoms may provide early clues suggesting COVID-19 infection and precipitate follow-up testing and precautions.

## CONCLUSION

Given the global pandemic, proper testing of individuals with suspected COVID-19 infection is crucial so that they can be identified and isolated. GI symptoms, such as anorexia, nausea, vomiting, diarrhea, and abdominal pain, even without the presence of respiratory symptoms, have been observed in patients with COVID-19. Therefore, COVID-19 infection should be considered for patients presenting with primarily GI symptoms.

## Notes

*This article meets the Accreditation Council for Graduate Medical Education and the American Board of Medical Specialties Maintenance of Certification competencies for Patient Care and Medical Knowledge.*
